# Beyond the Fever: A Serial Report on Moderate to Severe Murine Typhus Cases and Diagnostic Hurdles in Indonesia

**DOI:** 10.3390/tropicalmed10080204

**Published:** 2025-07-23

**Authors:** Velma Herwanto, Sandra Utami Widiastuti, Gunawan, Khie Chen Lie

**Affiliations:** 1 Department of Internal Medicine, Siloam Hospitals Kebon Jeruk, Jakarta 11530, Indonesia; sandra.utami@siloamhospitals.com; 2 Division of Internal Medicine, Faculty of Medicine, Universitas Tarumanagara, Jakarta 11440, Indonesia; 3 Department of Internal Medicine, Medistra Hospital, Jakarta 12950, Indonesia; dr_gunawan@medistra.com; 4 Division of Tropical Medicine and Infectious Diseases, Department of Internal Medicine, Faculty of Medicine, Universitas Indonesia/Cipto Mangunkusumo National General Hospital, Jakarta 10430, Indonesia; khie.chien@ui.ac.id

**Keywords:** murine typhus, rickettsiosis, acute febrile illness, nucleic acid amplification test, serology, doxycycline

## Abstract

(1) Background: Murine typhus, caused by *Rickettsia typhi*, is a neglected rickettsial disease and an underdiagnosed cause of acute febrile illness (AFI), particularly in endemic regions such as Indonesia. (2) Case description: We report a case series of four patients presenting with AFI of less than seven days in duration. Three patients were admitted with moderate disease, while one presented with septic shock with the macrophage activation-like syndrome (MALS) phenotype. Common clinical features included myalgia and headache; additional symptoms included cough, sore throat, and abdominal pain. Laboratory findings revealed bicytopenia, elevated transaminases, and raised inflammatory and bacterial infection markers. Common tropical infections—dengue, typhoid fever, and leptospirosis—and other potential sources of infection were excluded early during hospitalization. Diagnosis was confirmed by nucleic acid amplification testing (NAAT), which detected *R. typhi* in all patients. Doxycycline was initiated following confirmation, leading to defervescence within 36–48 h. (3) Conclusions: Murine typhus remains an underrecognized cause of febrile illness in Indonesia. In the near future, the inclusion of rickettsial testing in the diagnostic protocol of AFI will be crucial, as it enables timely administration of effective, low-cost treatment.

## 1. Introduction

Murine typhus is a rickettsial disease caused by the bacterium *Rickettsia typhi*. It is primarily transmitted to humans by rat fleas *Xenopsylla cheopsis*. Rickettsioses, including murine typhus, are among the most common causes of acute febrile illness (AFI) both globally and in Indonesia, yet they remain significantly underdiagnosed. In the AFIRE study, murine typhus was identified as the third most frequent cause of acute fever—following dengue virus and *Salmonella* spp. infections. However, none of the cases were diagnosed during hospitalization [1]. This underrecognition is largely due to overlapping clinical features with more common infections, as well as the limited availability of specific diagnostic tools [2].

Murine typhus typically presents as a mild illness characterized by abrupt fever and viral-like symptoms. However, a systematic review reported that severe disease with organ dysfunction can occur in approximately 3.8% to 28% of cases, potentially leading to intensive care unit admission or death, often due to delayed treatment [3].

Here, we present a series of murine typhus cases, initially presenting as acute fever, which were diagnosed late. The patients exhibited a spectrum of clinical manifestations ranging from mild illness to severe sepsis.

## 2. Case Illustration

This case series included four patients admitted with AFI lasting 4 to 7 days to two private hospitals in Jakarta, Indonesia, between April 2023 and October 2024. All patients exhibited a remittent fever pattern, which temporarily subsided with paracetamol administration, but recurred within several hours. Patient characteristics are summarized in Table 1.

Generalized myalgia was consistently reported across all cases, accompanying fever. Some patients also experienced a non-productive cough and headache. However, none of the patients exhibited symptoms that clearly indicated a specific source of infection such as lower respiratory tract symptoms, abdominal complaints, localized skin lesions, or urinary tract symptoms. Physical examinations were unremarkable, with no rash or eschar observed. Despite initial empirical treatments—targeting clinically suspected typhoid fever in Case 1 and 4 and pneumonia in Case 2 and 3—fever persisted in all patients for 3–4 days after admission, prompting consultations with infectious disease specialists.

One patient presented with a more severe clinical course. She was admitted on the seventh day of fever, initially reporting only myalgia. She had experienced mild diarrhea five days earlier, which had resolved within three days. Upon physical examination, she was hypotensive despite fluid resuscitation, tachycardic with weak peripheral pulses, exhibited cold extremities, and had mildly icteric sclerae. Laboratory investigations revealed anemia (hemoglobin 9.6 g/dL), leukocytosis (10,600/mm^3^), elevated creatinine (1.64 mg/dL), hyperbilirubinemia (2.23 mg/dL), and markedly elevated levels of procalcitonin and C-reactive protein/CRP (2.94 ng/mL and 203.9 mg/L, respectively). Abdominal ultrasonography revealed gallbladder sludge without signs of cholecystitis, a finding confirmed by magnetic resonance cholangiopancreatography (MRCP). The patient met the criteria for septic shock with a SOFA score of 8, without an identifiable source of infection. Subsequent evaluations showed a ferritin level of 14,578 ng/dL, a nadir platelet count of 112,000/mm^3^, INR of 1.14, and D-dimer level of 6100 µg/L, findings suggestive of a macrophage activation-like syndrome (MALS) phenotype in the context of sepsis.

The route of infection was unclear in all cases. Only one patient (Case 2) reported probable contact with rats, as he worked in a cluttered workshop where rats were frequently seen. Two patients (Cases 1 and 3) had pet dogs at home, and one of them (Case 1) had recently returned from a 7-day trip to Singapore, developing fever 5 days after his return. The most severely ill patient (Case 4) had no known exposure to animals or recent travel.

Comprehensive diagnostic workups had been performed to rule out more common causes of AFI. All four patients tested negative for dengue (NS1 antigen and anti-dengue serology), typhoid fever (anti-Salmonella IgM), malaria (rapid antigen and blood smear), and leptospirosis (nucleic acid amplification test/NAAT or anti-Leptospira IgM). Blood cultures remained sterile in all cases. Additional tests, including Cytomegalovirus (CMV) and Epstein−Barr virus (EBV) NAAT in Cases 1, 2, and 4, and multiplex NAATs for respiratory pathogens in Case 3, yielded no positive findings.

**Figure 1:**
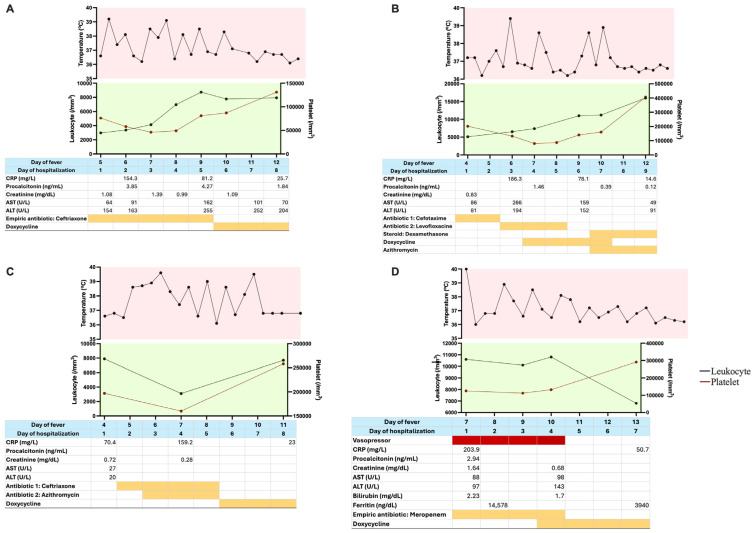
Serial clinical and laboratory data of the patients. (**A**) Case 1; (**B**) Case 2; (**C**) Case 3; (**D**) Case 4. Daily trends in body temperature, leukocyte count, platelet count, CRP, procalcitonin, creatinine, AST, and ALT are shown. Empirical antibiotic regimens and timing of clinical response to doxycycline were also indicated, illustrating disease progression and therapeutic outcomes. Red highlight: supportive therapy; orange highlight: antibiotic therapy.

Figure 1 presented the day-to-day clinical and laboratory data of the four cases. A consistent pattern was observed across patients, characterized by leukopenia and thrombocytopenia, with nadirs typically occurring between days 6 and 7 of illness. In patients without marked cytopenias, the leukocyte and platelet counts remained at the lower limits of the normal range. Both parameters improved in parallel with defervescence following the initiation of appropriate antimicrobial therapy.

All patients exhibited CRP levels exceeding 100 mg/L, consistent with a bacterial etiology. However, procalcitonin levels were only mildly elevated and did not correlate with the degree of CRP elevation. Transient elevations in transaminases and serum creatinine were noted in some patients, with normalization observed as clinical conditions improved. Peak transaminase levels also tended to occur around days 6 to 7 of fever.

Imaging studies were conducted to evaluate possible sources of infection. Chest imaging, either plain radiograph or CT, and abdominal imaging, either ultrasound or CT, were performed. In three patients, hepatosplenomegaly was observed without focal lesions. Pulmonary imaging revealed patterns consistent with pneumonia—either infiltrates or consolidations, with or without ground-glass opacities—in all four patients.

Murine typhus was diagnosed based on NAAT analysis of blood samples sent to a referral university laboratory, after the exclusion of other common infectious etiologies. All samples tested positive for *Rickettsia typhi*, except for one (Case 2), which was also positive for *Rickettsia* spp.—this was the only patient with likely rodent exposure.

Empirical antibiotic therapy was initiated upon admission: ceftriaxone for Cases 1 and 3, levofloxacine for Case 2, azithromycin for Case 3, and meropenem for Cases 3 and 4. These treatments resulted in no clinical improvement and no reduction in CRP or procalcitonin levels. Following confirmation of rickettsial infection, all patients were started on doxycycline 100 mg every 12 h, and previous antibiotics were discontinued. Doxycycline was initiated on hospital days 4 to 6. Fever resolved within 36–48 h of doxycycline initiation. The antibiotic was continued for 7 to 10 days on an outpatient basis in all cases except Case 2.

In Case 2, although fever initially subsided within 24 h of doxycycline initiation, it recurred the following day, accompanied by a maculopapular rash localized to the chest and back. A diagnosis of drug-induced exanthema, likely attributable to doxycycline, was created. The antibiotic regimen was changed to azithromycin 500 mg once daily, combined with oral dexamethasone. Fever resolved within 24 h of the new regimen, along with gradual improvement of the rash. Azithromycin was continued for four days, and corticosteroids were tapered over a seven-day course.

The most severely ill patient (Case 4) required norepinephrine due to septic shock. Doxycycline was initiated on day 10 of illness, after which hypotension, fever, bilirubin, and creatinine levels all improved significantly within 36–48 h. She gradually stabilized and was weaned off vasopressor support.

All four patients were discharged in a good clinical condition and remained well at follow-up, up to the time of manuscript preparation.

## 3. Discussion

We present a case series of murine typhus in patients hospitalized with AFI. All patients reported persistent fever at the time of admission; however, the diagnosis was not established until days 4–5 of hospitalization. Three patients exhibited moderate clinical severity, while one presented with septic shock.

Murine typhus, caused by *Rickettsia typhi*, remains a common but underrecognized cause of AFI in endemic regions, including Indonesia. A large multicenter study across eight tertiary hospitals identified murine typhus in 10.6% of AFI cases (103/975), making it the third most common etiology. Notably, none of these cases were diagnosed during hospitalization; all were retrospectively confirmed via NAAT and serology from stored samples. Misdiagnoses included dengue, typhoid fever, leptospirosis, sepsis, and respiratory and urinary tract infections, or remain undiagnosed [1,4].

In our series, murine typhus was considered only after more prevalent infections—such as dengue, typhoid, leptospirosis, and sepsis—had been excluded. There are currently no standardized clinical criteria for suspected or probable rickettsial infections. In Indonesia, testing for rickettsiae is typically pursued only after empirical therapy fails or other diagnoses are ruled out, contributing to diagnostic delays and frequent underrecognition.

All patients in our cohort presented with fever, myalgia, and headache—findings consistent with the AFIRE study, in which headache and myalgia were present in 69% and 28% of murine typhus cases, respectively. Respiratory symptoms, including cough and radiographic fibroinfiltrates, were also observed. These are likely due to capillary leakage from *R. typhi*-induced endothelial injury [5] and resolved following antimicrobial therapy.

The most severely ill patient had mild diarrhea and abdominal pain—gastrointestinal (GI) symptoms are reported in 18–19% of cases [5]. Other GI symptoms (e.g., anorexia, nausea, and vomiting) were absent. No patients exhibited rash or eschar. Rash is often underreported in murine typhus and occurred in only 17% of cases in the AFIRE study [4,6]. The absence of rash, eschar, and known exposure history made clinical suspicion more challenging [7].

Flea-borne transmission via rat or cat fleas is typical [8], yet only two patients had possible exposure—one through occupational contact with rats and another through gardening. The remaining two patients had pet dogs, which, while less commonly recognized, have been reported in the literature as potential carriers of *X. cheopis* [9]. Although Singapore is not considered endemic for murine typhus, a seroprevalence study found that approximately one-third of rodents in Singapore were seropositive for *R. typhi* [10], suggesting possible environmental exposure. Interestingly, Case 2 tested positive for other Rickettsia species (*R. prowazekii* or spotted fever group rickettsiae), possibly reflecting exposure to multiple vectors beyond flea rat.

Liver involvement was evident in all patients, with elevated transaminases and bilirubin levels. This is consistent with *R. typhi*-induced periportal inflammation and focal hepatitis without direct hepatocyte invasion [11]. In the AFIRE cohort, 77% had elevated liver enzymes and 21% had hyperbilirubinemia [4]; these abnormalities resolved with treatment.

Hematologic findings included leukopenia and thrombocytopenia, consistent with prior reports from Indonesia (12% and 80%, respectively) [12]. CRP and procalcitonin levels were elevated, mimicking bacterial sepsis. However, most rickettsial cases demonstrate CRP < 150 mg/L and procalcitonin < 2 ng/mL [13], which may aid in distinguishing them from severe bacterial infections, where these inflammatory markers are typically more markedly elevated.

Complications included acute kidney injury and sepsis. In published studies, these occur in approximately 10% and 6% of murine typhus cases, respectively [4,12]. One patient (Case 4) fulfilled criteria for MALS, a sepsis phenotype marked by ferritin > 4420 ng/mL and associated with a poor prognosis [14]. This patient improved with timely doxycycline and supportive care, including reduction in ferritin alongside clinical recovery. Fulminant murine typhus with MALS has been previously documented [15].

Given the clinical manifestations described in this case series, certain clinical features may aid in differentiating murine typhus from other tropical febrile illnesses. Fever in murine typhus typically persists beyond five days, unlike dengue, which often resolves by this point. Typhoid fever presents with stepwise fever and more prominent GI symptoms. Murine typhus, by contrast, usually presents with abrupt fever onset. Leptospirosis is more likely to produce marked jaundice and significant hyperbilirubinemia. Laboratory findings such as leukopenia, thrombocytopenia, elevated CRP, mildly increased procalcitonin, and, less commonly, elevated liver enzymes or acute kidney injury may further support the diagnosis of murine typhus.

All patients responded rapidly to doxycycline, with defervescence typically within 1–2 days. Initial empirical treatments included ceftriaxone, based on presumptive diagnoses of typhoid fever or leptospirosis. The temporal association between doxycycline initiation and clinical improvement supports the diagnosis of murine typhus. Although delayed defervescence can occur, this is thought to be due to host factors rather than antimicrobial resistance, as doxycycline resistance in *R. typhi* remains unconfirmed [16,17]. In Case 2, doxycycline was discontinued due to a suspected drug-induced exanthem, which obscured the evaluation of fever resolution following its administration. The antibiotic was subsequently switched to azithromycin, which led to clinical improvement and resolution of fever. Azithromycin has been studied as an effective alternative to doxycycline, although it may be associated with slower fever resolution [18].

The prognosis for murine typhus is generally favorable. A systematic review estimated an untreated mortality rate of 0.4%, though rates may be higher in resource-limited settings [3,4]. Poor outcomes are associated with older age, comorbidities, and severe presentations such as septic shock—as seen in our most critical case [19]. Nonetheless, early recognition and appropriate therapy can markedly reduce morbidity and mortality.

In conclusion, we presented a series of murine typhus cases admitted with AFI of less than seven days in duration. Diagnosis was established using NAAT on blood samples. Three patients presented with mild to moderate illness, while one developed severe sepsis. All patients improved with appropriate antimicrobial therapy.

A major challenge in case management was the initial use of empirical antibiotics targeting alternative etiologies such as typhoid fever and leptospirosis. One patient also received glucocorticoids for a presumed drug reaction. Despite these interventions, clinical improvement only occurred after doxycycline was initiated, highlighting its therapeutic efficacy.

A key limitation is the restricted access to diagnostic testing. At the time of this report, NAAT for rickettsia was available at only one academic center in Jakarta and had only recently become commercially accessible. Current assays detect *R. typhi* and *Rickettsia* spp., but not *Orientia tsutsugamushi*, the agent of scrub typhus, which is also endemic in Indonesia and accounts for approximately 8.8% of AFI cases [20]. Moreover, the sensitivity of NAAT alone is limited; one study reported a detection rate as low as 30%. Consequently, patients testing negative using NAAT alone may be misclassified, leading to an underdiagnosis of murine typhus. To improve diagnostic accuracy, a combination of NAAT and serological testing is recommended [21]. However, serologic testing is largely unavailable domestically, with some private laboratories outsourcing samples abroad. This lack of diagnostic capacity hinders the timely identification and treatment of rickettsial diseases, despite the availability of effective therapy.

Based on our findings, we propose the following recommendations to improve the recognition and management of murine typhus in endemic areas: (1) Establish standardized surveillance systems and expand availability of affordable, locally validated diagnostic tools for murine typhus and other rickettsial diseases in Indonesia and the broader Southeast Asian region. (2) Integrate rickettsial testing into routine AFI diagnostic algorithms, especially in regions with known endemicity, as such testing may not be a priority in non-endemic settings (see proposed AFI algorithm in Supplementary Material). (3) Consider empirical doxycycline in febrile patients with clinical features suggestive of murine typhus, particularly when diagnostic tools are limited for frontline clinicians. (4) Improve access to intravenous doxycycline, currently unavailable in Indonesia, which is essential for patients with severe disease or those unable to take oral medications.

## Figures and Tables

**Table 1:** Patients’ characteristics.

Clinical Data	Case 1	Case 2	Case 3	Case 4
Demography				
Sex	Male	Male	Female	Female
Age, y.o.	29	57	35	63
Comorbid condition(s)	None	Dyslipidemia, hypertension, prediabetic	History of pulmonary tuberculosis 5 years before	Hypertension, prediabetes
History				
Suspected transmission route	History of travelling to Singapore 5 days before; had pet dogs	Rats at cluttered shop	Contact with pet dog	Gardening hobby
Day of fever	5	4	4	7
Myalgia/arthralgia	Yes	Yes	Yes	Yes
Headache	Yes	Yes	Yes	Yes
Cough	No	Yes	Yes, productive	Yes
Abdominal pain	No	No	No	Yes
Other symptoms	None	None	Sore throat	Asthenia, brownish urine
Physical examination				
Highest temperature, °C	39.1	39.2	39.5	41
Rash/eschar	None	Rash, cannot be differentiated from drug-induced allergic reaction	None	None
Imaging Findings				
Chest X-ray/CT	Minimal fibroinfiltrate on bilateral lower lung lobes	Focal consolidation with air bronchogram, with ground glass opacification and fibrosis on one segment	Focal consolidation surrounded with ground glass opacification with air bronchogram on the basal	None
Abdominal ultrasonography/CT	Hepatosplenomegaly	Hepatosplenomegaly	No abnormality	Mild fatty liver, nephrolithiasis
Laboratory Findings				
Lowest leukocyte count, /mm^3^	2980	5100	3100	6800
Lowest platelet count, /mm^3^	46,000	80,000	160,000	112,00
Highest AST, U/L	162	266	27	143
Highest ALT, U/L	255	194	20	98
Highest creatinine, mg/dL	1.39	0.83	0.72	1.64
Highest CRP, mg/L	154.3	186.3	159.2	203.9
Highest procalcitonin, ng/mL	4.27	1.46	0.28	2.94
PCR Rickettsia	*R. typhi*	*R. typhi* and *R.* spp.	*R. typhi*	*R. typhi*
Response to Therapy				
Time of fever defervescence following doxycycline, hours	36	36	36	48

## Data Availability

No new data were created or analyzed in this study. Data sharing is not applicable to this article.

## Supplementary Materials

The following supporting information can be downloaded at: https://www.mdpi.com/article/10.3390/tropicalmed10080204/s1. Figure S1: Proposed diagnostic algorithm for acute febrile illness (AFI) in Indonesia or other countries with similar patterns of endemic infectious diseases [1,22].

## Abbreviations

The following abbreviations are used in this manuscript:

AFI	Acute febrile illness
CRP	C-reactive protein
MALS	Macrophage activation-like syndrome
NAAT	Nucleic acid amplification test/testing
