# Supplementary material for: Beyond the Fever: A Serial Report on Moderate to Severe Murine Typhus Cases and Diagnostic Hurdles in Indonesia
Source: Trop Med Infect Dis. 2025 Jul 23;10(8):204. doi: 10.3390/tropicalmed10080204 (PMC12390629; doi:10.3390/tropicalmed10080204)
Supplement: Supplementary file 1 [file tropicalmed-10-00204-s001.zip › tropicalmed-3734747-supplementary.pdf]

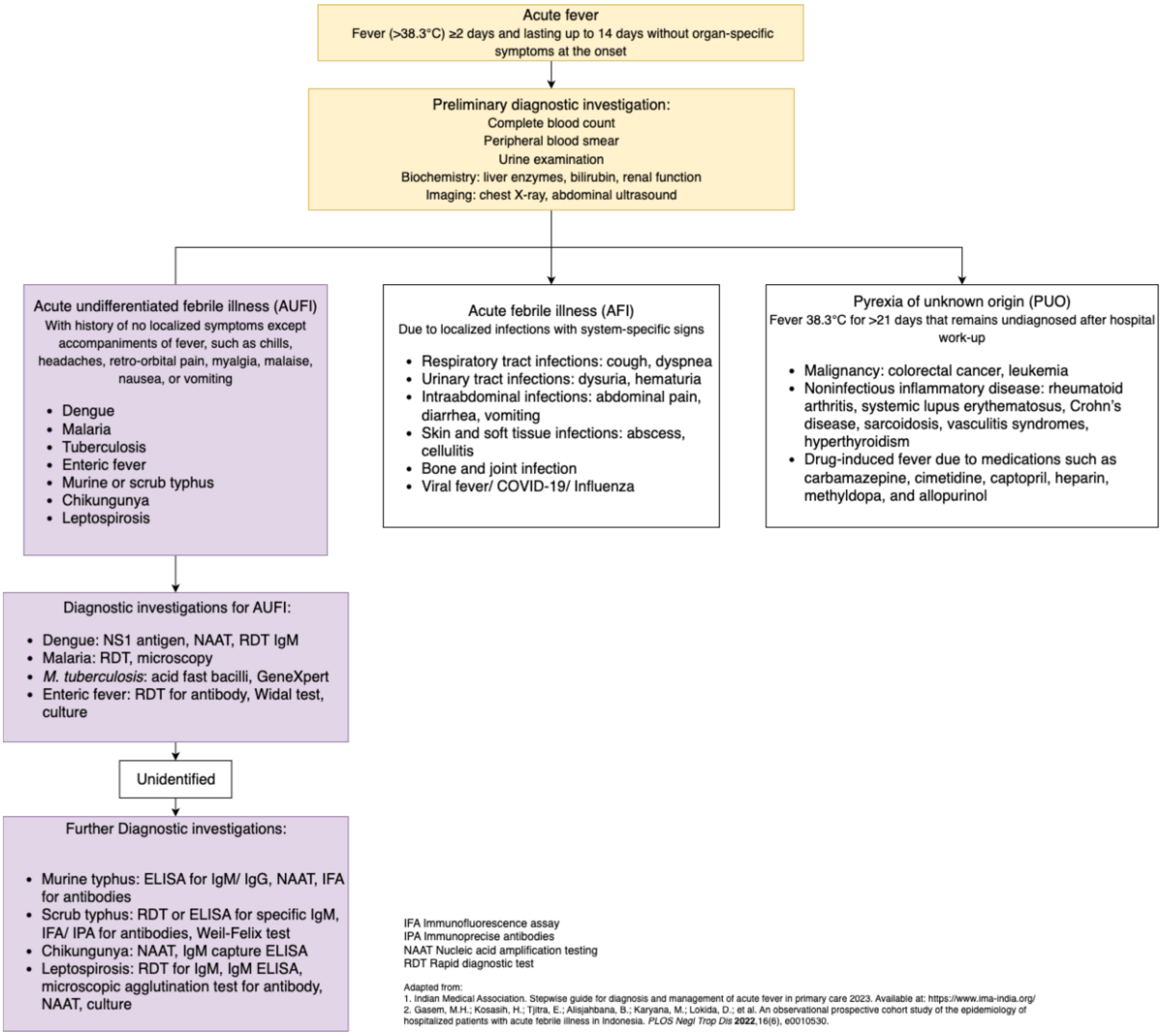

Figure S1. Proposed diagnostic algorithm for acute febrile illness (AFI) in Indonesia or other countries with similar patterns of endemic infectious diseases
